# A morphological analysis of calcium hydroxylapatite and poly‐l‐lactic acid biostimulator particles

**DOI:** 10.1111/srt.13764

**Published:** 2024-06-09

**Authors:** Alec D. McCarthy, Christian Hartmann, Alan Durkin, Shatil Shahriar, Saami Khalifian, Jingwei Xie

**Affiliations:** ^1^ Medical Affairs Merz Aesthetics Raleigh North Carolina USA; ^2^ R&D Merz Aesthetics GmbH Frankfurt Germany; ^3^ Ocean Drive Plastic Surgery Vero Beach Florida USA; ^4^ University of Nebraska Medical Center Omaha Nebraska USA; ^5^ SOM Aesthetics Encinitas California USA

**Keywords:** CaHA, calcium hydroxylapatite, PLLA, poly‐l‐lactic acid, radiesse, sculptra

## Abstract

Injectable fillers, pivotal in aesthetic medicine, have evolved significantly with recent trends favoring biostimulators like calcium hydroxylapatite (CaHA‐CMC; Radiesse, Merz Aesthetics, Raleigh, NC) and poly‐l‐lactic acid (PLLA; Sculptra Aesthetics, Galderma, Dallas, TX). This study aims to compare the particle morphology of these two injectables and examine its potential clinical implications. Utilizing advanced light and scanning electron microscopy techniques, the physical characteristics of CaHA‐CMC and PLLA particles were analyzed, including shape, size, circularity, roundness, aspect ratio, and quantity of phagocytosable particles. The findings reveal several morphological contrasts: CaHA‐CMC particles exhibited a smooth, homogenous, spherical morphology with diameters predominantly ranging between 20 and 45 µm, while PLLA particles varied considerably in shape and size, appearing as micro flakes ranging from 2 to 150 µm in major axis length. The circularity and roundness of CaHA‐CMC particles were significantly higher compared to PLLA, indicating a more uniform shape. Aspect ratio analysis further underscored these differences, with CaHA‐CMC particles showing a closer resemblance to circles, unlike the more oblong PLLA particles. Quantification of the phagocytosable content of both injectables revealed a higher percentage of phagocytosable particles in PLLA. These morphological distinctions may influence the tissue response to each treatment. CaHA‐CMC's uniform, spherical particles may result in reduced inflammatory cell recruitment, whereas PLLA's heterogeneous particle morphology may evoke a more pronounced inflammatory response.

## 1 INTRODUCTION

Injectable fillers have long been used for soft tissue augmentation, particularly for aesthetic treatment modalities, and have continued to grow in popularity.^1^ Historically, most injectable fillers were hyaluronic acid (HA) gels that yielded immediate volumization and gradually decreased as the gel was metabolized. While this has been an effective method for volumization, advancements in regenerative medicine and biomaterial engineering introduced a new class of fillers that contain particles capable of regenerating portions of the extracellular matrix (ECM).^2^ Two of the most popular regenerative fillers are calcium hydroxylapatite (CaHA‐CMC; Radiesse, Merz Aesthetics, Raleigh, NC) and poly‐l‐lactic acid (PLLA; Sculptra Aesthetics, Galderma, Dallas, TX). CaHA‐CMC is composed of 70% v/v carboxymethylcellulose gel and 30% v/v calcium hydroxylapatite (CaHA) microspheres (25–45 µm in diameter) and has been shown to regenerate collagen 1 and 3, elastin, and proteoglycans via mechanical activation of fibroblasts (mechanotransduction).^3^, ^4^ It has also been shown to recover fibroblast contractile strength, modulate skin smoothness, and restore the structure and function of aged skin with minimal immune cell involvement.^5^, ^6^, ^7^ CaHA‐CMC is routinely injected in its undiluted form for volumetric correction and in dilute or hyperdilute configurations for biostimulation.^8^, ^9^, ^10^

PLLA is composed of 10–200 µm diameter poly‐l‐lactic acid micro particles that are reconstituted with water or saline.^11^ PLLA has been shown to regenerate collagen 1 as well as improve the appearance of the skin.^12^, ^13^ Stein et. al. showed that PLLA particles were encapsulated rather by the less organized collagen type III than type I.^14^ Data suggests that the mechanism of action of PLLA relies on the involvement of the immune system for neocollagenesis, a distinctly different mechanism of action compared to CaHA‐CMC.^11^, ^15^, ^16^ Previous studies indicate the particles that compose PLLA are heterogeneous and resemble flakes, rather than smooth microspheres.^17^ This irregular morphology may compromise the direct filling effect of PLLA‐SCA, while simultaneously eliciting a more pronounced inflammatory response due to the presence of small fragmented particles and sharp particle edges.^14^ Moreover, recent studies have highlighted PLLA‐SCA's propensity to elicit increased inflammatory cell response and activation of pro‐inflammatory cytokines, particularly M1 and M2 macrophages.^16^, ^18^ Confocal imaging has provided insights into the formation of macrophage and giant cell capsules at the surface of PLLA‐SCA particles, further underscoring its immunostimulatory effects.^19^

The current study compares the morphology of CaHA‐CMC and PLLA‐SCA particles and analyzes key morphologic features including circularity, roundness, aspect ratio (AR), and phagocytosable content using both light microscopy and scanning electron microscopy.

## 2 MATERIALS AND METHODS

### 2.1 Materials

CaHA (Radiesse, Merz Aesthetics, Raleigh, NC, USA) and PLLA‐SCA (Sculptra, Galderma, Fort Worth, TX, USA) were utilized for this study. The PLLA was reconstituted following the instructions for use with a 5 mL reconstitution recommendation. The PLLA‐SCA vial was shaken vigorously by hand and placed on a shake plate for 24 h to undergo thorough agitation. Prior to use, the vial of PLLA‐SCA was manually agitated with gentle shaking. The CaHA was diluted with 3.0 mL of sterile water using a male‐to‐male Luer lock adaptor and was homogenized with 20 back‐and‐forth strokes. Notably, since the CMC gel component in CaHA‐CMC is not crosslinked, dilutions past 1:1 (w/v) result in a near‐zero elastic moduli suspension in which the CMC gel is entirely dispersed.^10^ In all cases, and for both products, a 26‐gauge needle was used during extrusion, consistent with the needle size recommended by both companies for their respective products.

### 2.2 Microscopy characterization

For scanning electron microscopy (SEM), CaHA microspheres and PLLA flakes were prepared and processed as described by Kunzler et al. 2023.^20^ In brief, microparticles of each biostimulator were dried and transferred to an SEM stage containing carbon tape. Samples were analyzed using a Phenom (PhenomWorld, Netherlands) pure electron microscope with a backscattered electron detector. Acceleration voltage was 5 kV, and the samples were sputtered with gold for increased resolution. For brightfield microscopy, isolated CaHA microspheres and PLLA flakes were dispersed on glass‐bottom Ibidi slides (µ‐slide, eight well chambers; Ibidi Germany) and images were acquired using an inverted Axio Observer 7 (Carl Zeiss, Germany) microscope.

### 2.3 Image analysis

**FIGURE 1:**
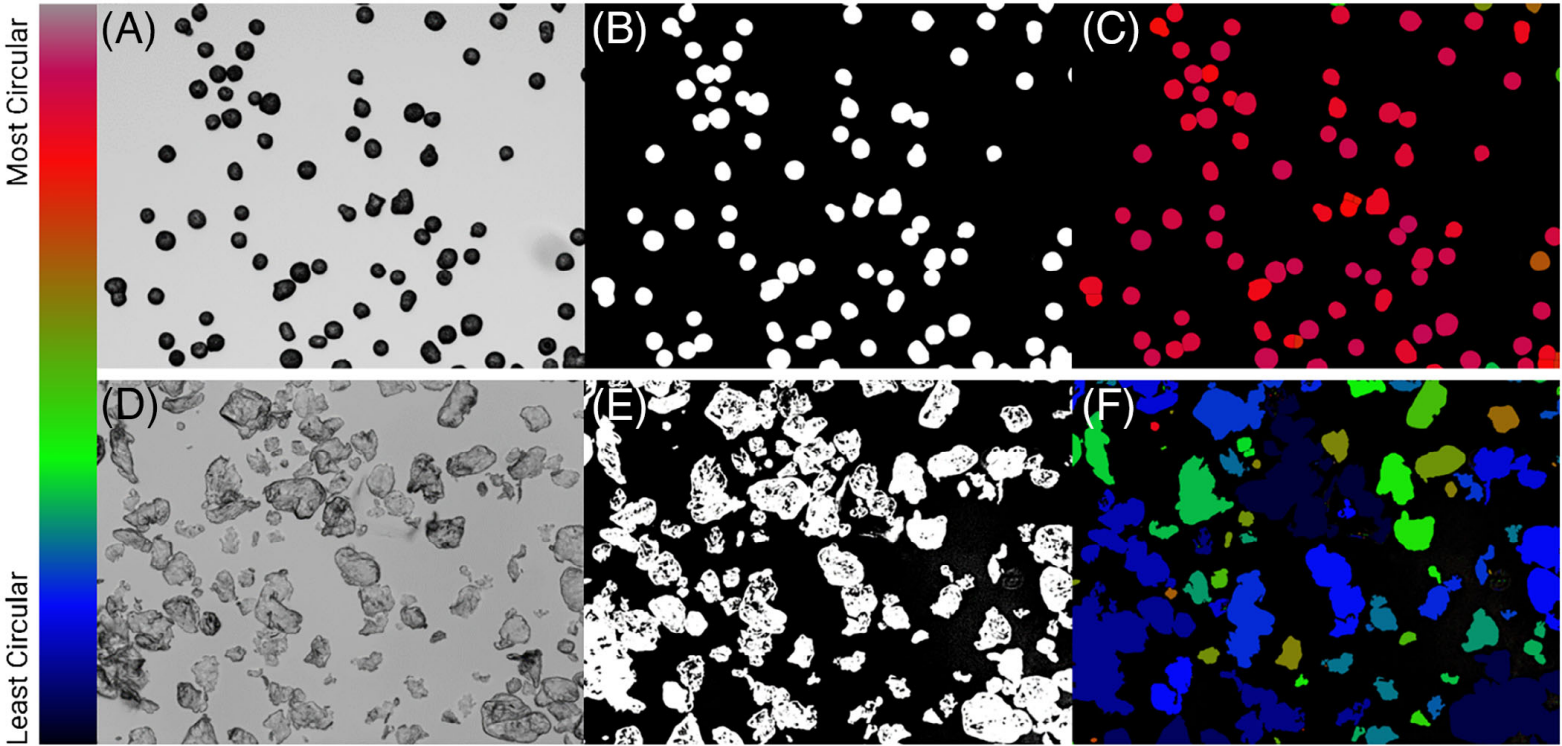
An example of the workflow for image analysis using ImageJ's biovoxxel plugin. (A) Standard brightfield image of calcium hydroxylapatite (CaHA) particles prior to analysis. (B) Shows the particles after applying a binary mask. (C) Shows the post watershed colorimetric heat map with pink showing perfect circularity. (D) Brightfield images of poly‐l‐lactic acid (PLLA) (E) after binary conversion and (F) after colorimetric shape mapping.

ImageJ FIJI was used for all image analyses. To measure roundness, circularity, and aspect ratio, the brightfield images were converted to 8‐bit, and a Huang threshold was applied so that individual particles were highlighted. Once the threshold was applied, the images had a binary hole‐fill applied, leaving only the outline of each particle. Once particles were filled, a watershed was applied to segment neighboring particles. Next, a shape descriptor map was utilized to generate a color map for circularity, roundness, aspect ratio, and so forth, using the BioVoxxl ImageJ Plugin. A sample workflow for measuring circularity is seen in Figure 1. To measure the quantity of phagocytosable material, a size filer was applied to the SEM images using ImageJ. Images were converted to binary and a particle analysis was carried out to exclude particles over a major axis length of 20 µmin diameter, based on the size put forth by Lemperle.^21^

### 2.4 Statistical analysis

A minimum of 150 particles/flakes of each material were analyzed. Descriptive statistics were computed, and results expressed as mean ± standard deviation. When appropriate, box‐and‐whisker plots were used with mean expressed as the central line and error bars representing the range of min to max. When appropriate, bar graphs were used with error bars representing the standard deviation. For both box‐and‐whisker and bar graphs, unpaired *t*‐tests were used to compute significance, defined as: not significant (ns), *p* > 0.05, *p < 0.05, **p < 0.01, ***p < 0.001, ****p < 0.0001. When appropriate, histograms were deployed to visualize value distributions. Histograms were created by applying bins to the data sets and a nonlinear line, least squares fit line was computed and smoothed using a second‐ordered smoothing. To test for significance between histograms, the Kolmogorov–Smirnov test was used. All data was graphed and analyzed in GraphPad Prism 9 for macOS (Version 10.2.1, GraphPad, San Diego, CA, USA) and all figures were created using BioRender (BioRender.com, Toronto, ON, CA).

## 3 RESULTS

**FIGURE 2:**
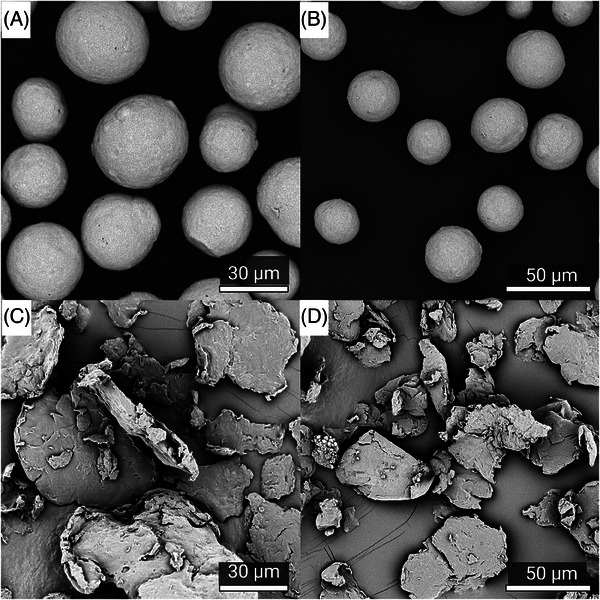
Scanning electron microscopy (SEM) images of calcium hydroxylapatite (CaHA‐CMC) (A and B) and PLLA (C and D) particles.

**FIGURE 3:**
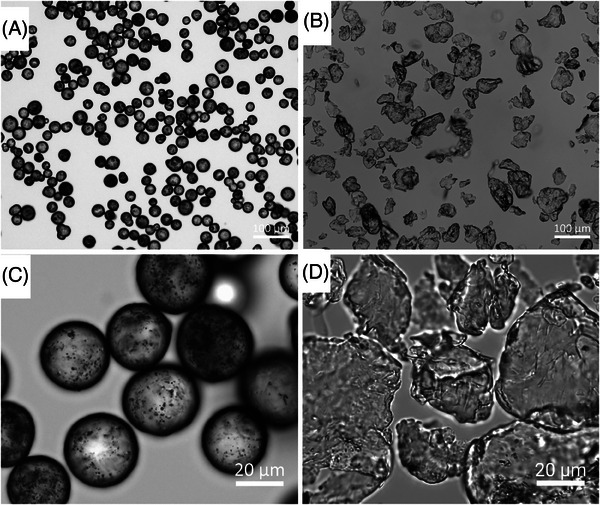
Brightfield images of calcium hydroxylapatite (CaHA‐CMC) (A and C) and poly‐l‐lactic acid (PLLA) (B and D) particles.

Visualization of the CaHA and PLLA components of both fillers reveal dramatic differences in morphology; CaHA appeared as smooth, homogenous microspheres ranging between 25 and 45 µm in diameter (Figure 2A,B), and the PLLA particles appeared as microflakes ranging from 2 to 150 µm in major axis length (Figure 2C,D). These findings were further verified under light microscopy, which showed uniform and highly spherical CaHA microspheres and PLLA microflakes (Figure 3).

**FIGURE 4:**
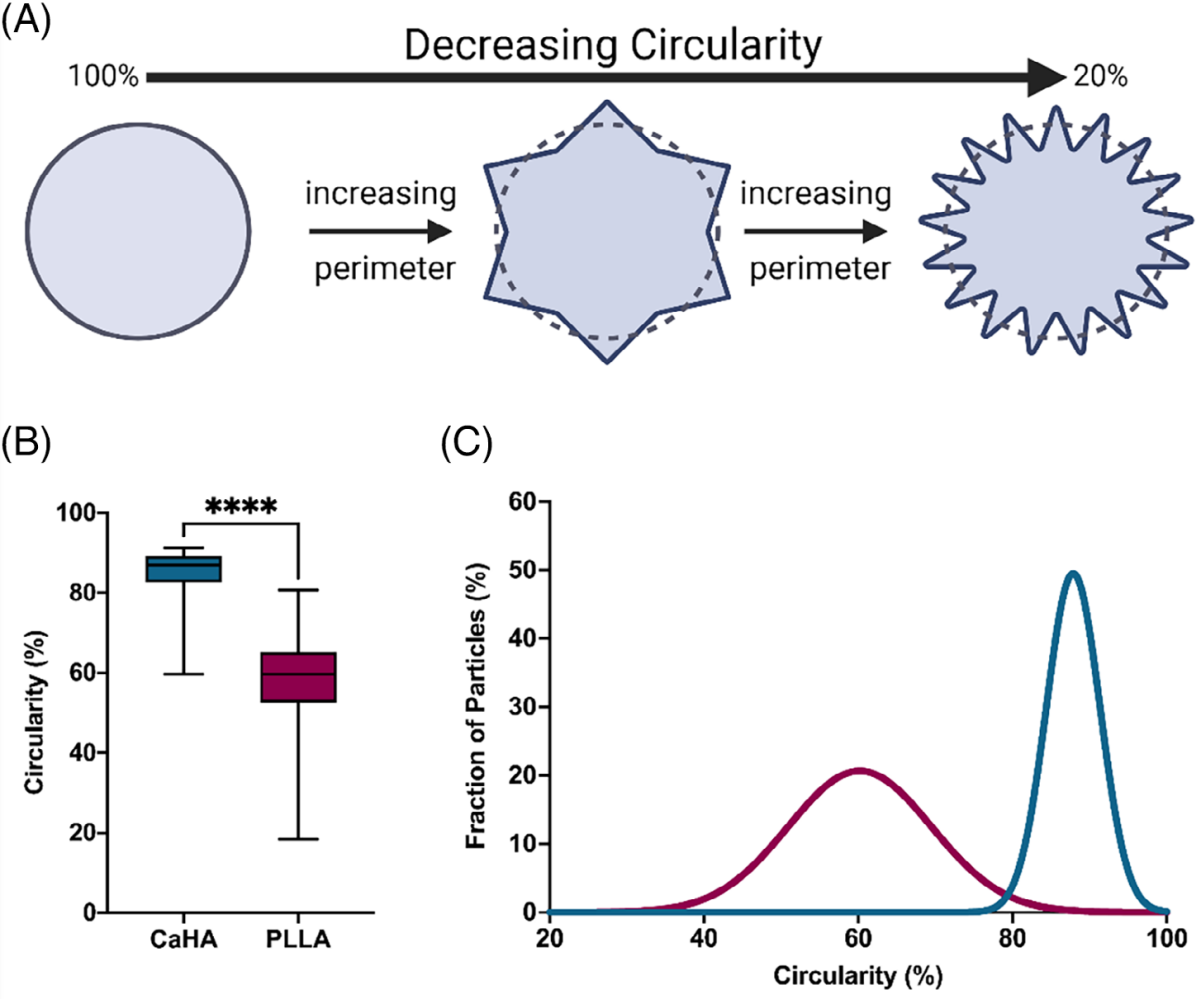
Circularity of calcium hydroxylapatite (CaHA‐CMC) and poly‐l‐lactic acid (PLLA) particles. (A) The quantification of circularity graphically illustrated, with a perfect circle measuring at 100% circularity. (B) Box‐and‐whisker plots of CaHA‐CMC and PLLA particle circularity and their (C) frequency distributions illustrating significantly different average circularities and significantly different frequency distributions.

Circularity specifically measures an object's boundary by comparing the object's actual perimeter (the length of its boundary) to the perimeter of a perfect circle with the same area (Figure 4A). Circular objects will have a circularity value closer to 1 (scaled to 100%), indicating their boundaries closely resemble a circle. Objects with irregular or complex boundaries will have lower circularity values, approaching 0 (scaled to 0%). The mean circularity of CaHA was observed to be 85.01 ± 0.056 with a maximum value of 91.30% and a minimum value of 59.70% (on a scale of 0%–100%). The mean circularity of PLLA‐SCA particles was observed to be 58.41% ± 0.11% with a maximum circularity of 80.70% and a minimum value of 18.40%. The difference in mean values varied significantly between the groups (*p* < 0.0001) (Figure 4B). The distribution in circularity shows a significantly tighter distribution for CaHA microspheres, with most falling between 85% and 90%. PLLA‐SCA microparticles had a broad distribution range between 55% and 70% (K‐S D = 0.8964, *p* < 0.0001) (Figure 4C).

**FIGURE 5:**
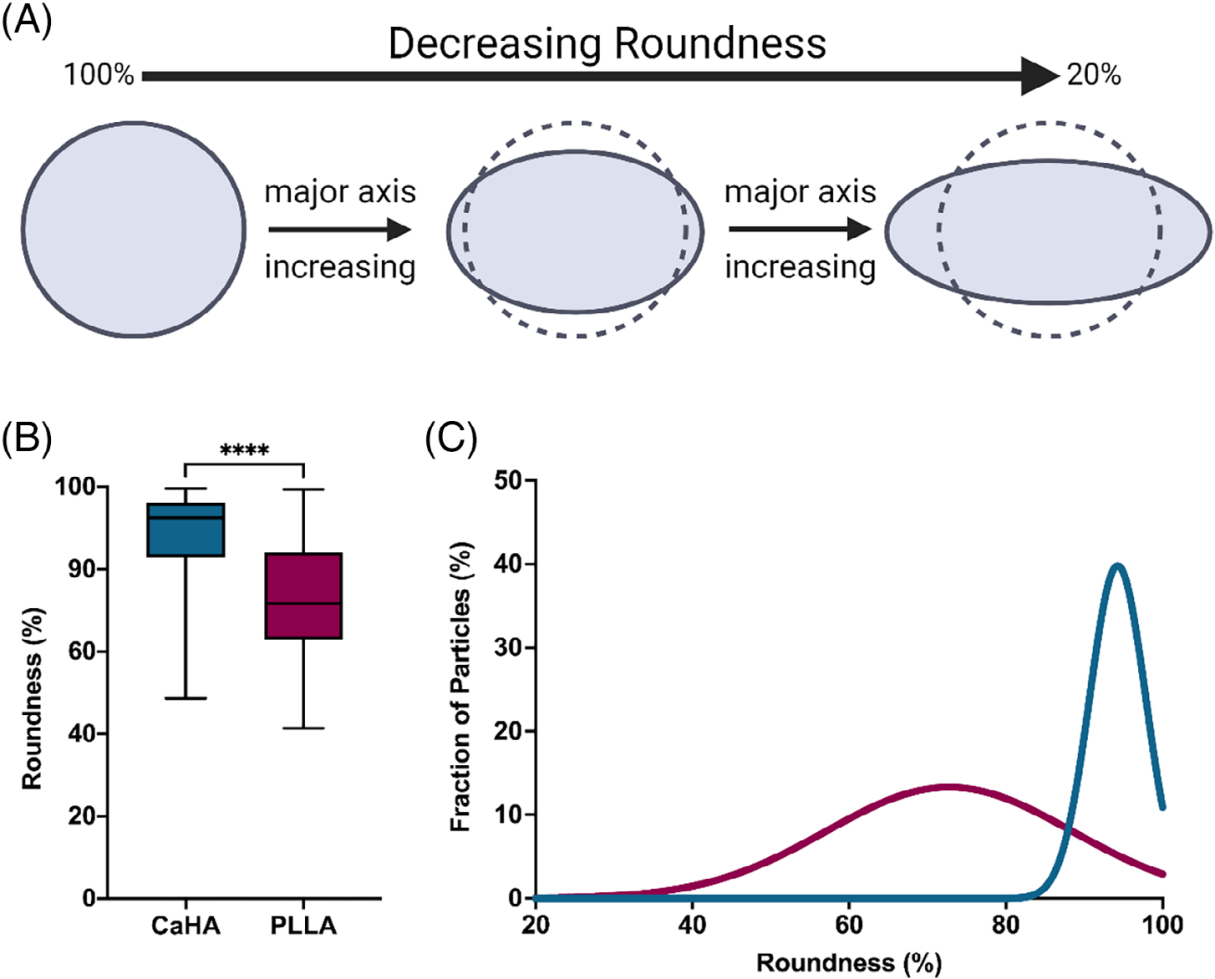
Roundness of calcium hydroxylapatite (CaHA‐CMC) and poly‐l‐lactic acid (PLLA) particles. (A) The quantification of roundness graphically illustrated, with a perfect circle measuring at 100% round. (B) Box‐and‐whisker plots of CaHA‐CMC and PLLA particle circularity and their (C) frequency distributions illustrating significantly different average roundness and significantly different frequency distributions.

While similar, roundness measurements differ from circularity measurements. Roundness is a measurement that assesses how close an object's shape is to a perfect circle by considering the ratio of the object's area to the area of the smallest enclosing circle (Figure 5A). In simpler terms, it evaluates how “round” an object appears. If the object is elongated or irregular, the roundness value will be closer to 0 (scaled to 0%). If the object is more circular in shape, the roundness value will be closer to 1 (scaled to 100%). CaHA particle roundness was measured to be 88.63% ± 0.10% while PLLA‐SCA particle roundness was measured to be 72.65% ± 13.21%. The difference in mean values varied significantly between the groups (*p* < 0.0001) (Figure 5B). Similarly, the frequency distributions vary significantly, with CaHA particles having a tight distribution between approximately 85%–100% and PLLA‐SCA having a broad distribution between around 65% and 85% (K‐S D = 0.5393, *p* < 0.0001) (Figure 5C).

**FIGURE 6:**
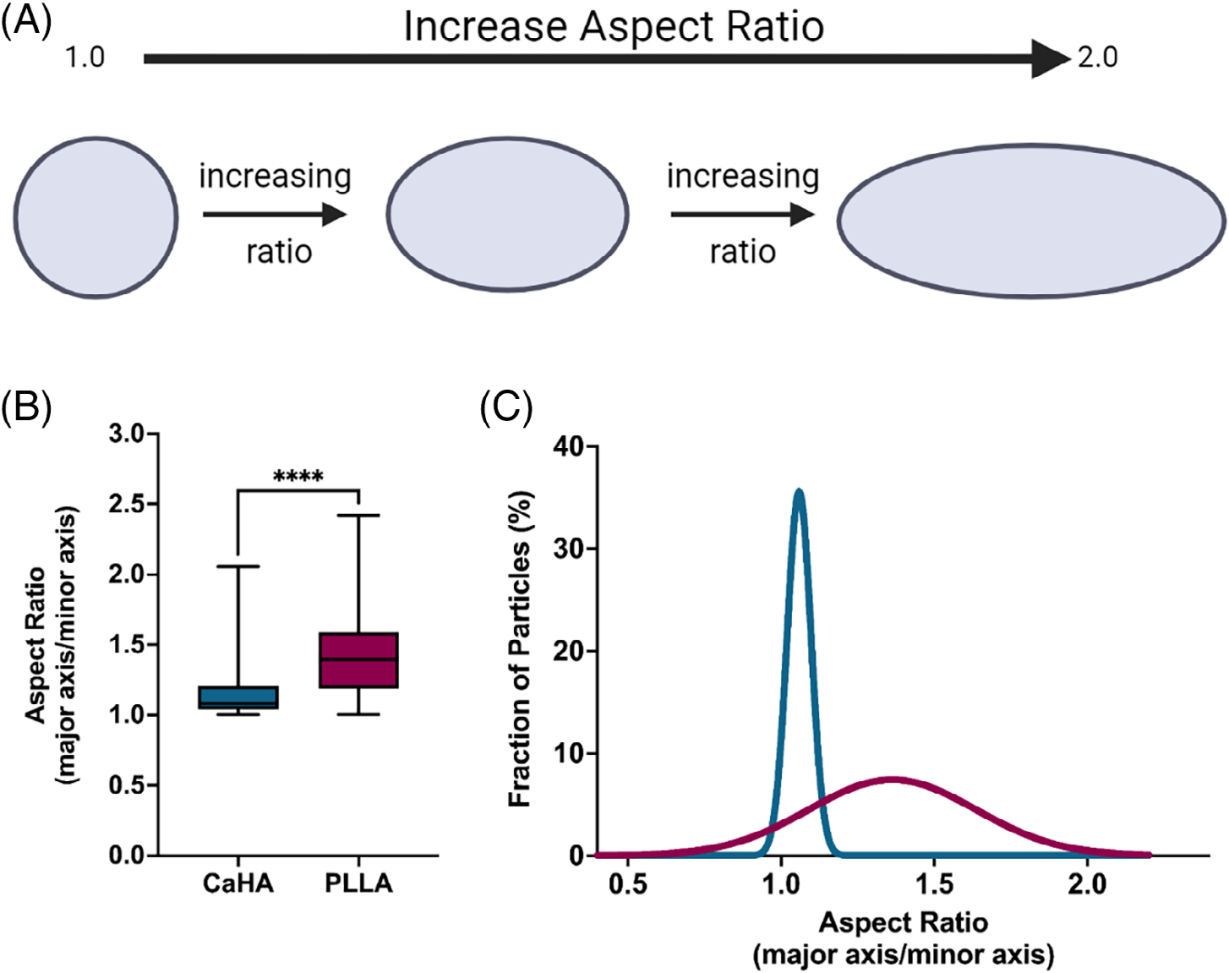
Aspect ratios of calcium hydroxylapatite (CaHA‐CMC) and poly‐l‐lactic acid (PLLA) particles. (A) The quantification of aspect ratio graphically illustrated, with a perfect circle measuring at 1.0 and increasing with axial elongation. (B) Box‐and‐whisker plots of CaHA‐CMC and PLLA particle aspect ratios and their (C) frequency distributions illustrating significantly different average aspect ratios and significantly different frequency distributions.

Another morphological feature, aspect ratio (AR), is a ratio of major axis length to minor axis length and provides insight into the axial proportionality of a particle. A perfect circle, for example, would have an AR of precisely 1, while an oval would have an AR greater than 1 (Figure 6A). AR quantification is unitless, starts at 1, and increases indefinitely based on axis length. CaHA particles are both round and circular and have an AR of 1.145 ± 0.153. In contrast, PLLA‐SCA particles have a significantly higher AR of 1.426 ± 0.279 (*p* < 0.0001) (Figure 6B). As evident in Figure 5A, the upper range of AR for PLLA‐SCA approaches 2, indicating most particles are oblong. The AR frequency distributions vary significantly, with CaHA particles having a smaller distribution range (AR 1.004 to 2.054 and PLLA‐SCA having a broad distribution (AR 1.006 to 2.418 (K‐S D = 0.9444, *p* < 0.0001) (Figure 6C).

**FIGURE 7:**
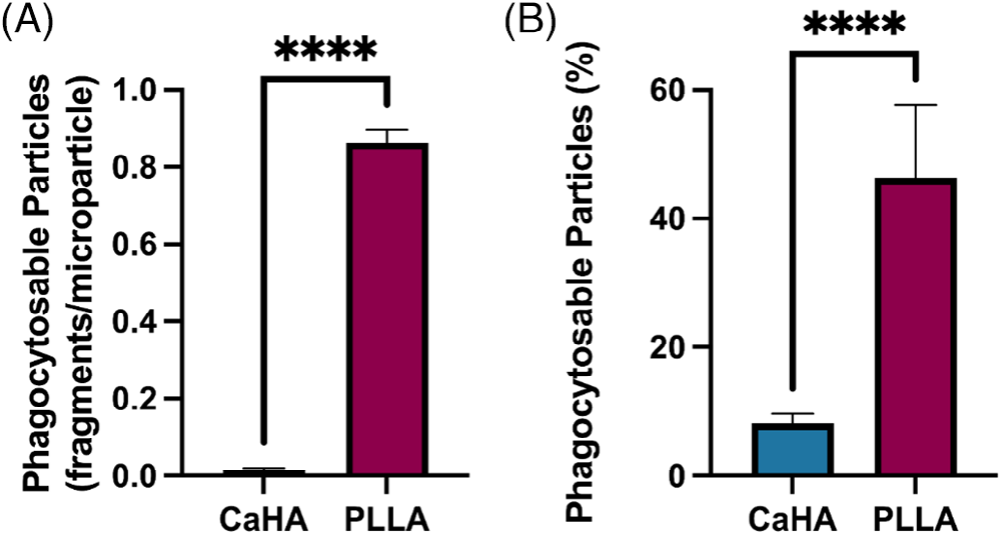
Differences in phagocytosable material in samples of calcium hydroxylapatite (CaHA‐CMC) and poly‐L‐lactic acid (PLLA) particles. (A) The ratio of phagocytosable particles (<20 µmin major axis) relative to total particles. (B) The percentage of total particles with major axes under 20 µmin length.

Phagocytosable content, measured as particles whose major axis diameter was under 20 µm as described by Lemperle et al., were quantified and compared.^21^ The percentage of phagocytosable particles (particles under 20 µm relative to the total particle count) in the CaHA samples was 8.20% ± 1.45% while the number of phagocytosable particles of PLLA‐SCA was 46.32% ± 11.33% (*p* < 0.0001) (Figure 7). A summary of morphological properties and their differences is available in Table 1.

**TABLE 1:** Average morphological properties of calcium hydroxylapatite (CaHA) and poly‐l‐lactic acid (PLLA) particles.

Material	Circularity	Roundness	Aspect ratio	Phagocytosable fragments (%)
CaHA	85	89	1.1	8
PLLA	58	73	1.4	46
*p* Value	<0.0001	<0.0001	<0.0001	<0.0001

## 4 DISCUSSION

The present study offers a comprehensive morphological comparison between CaHA‐CMC and PLLA‐SCA, shedding light on the distinctive characteristics of these widely used regenerative fillers. Our findings demonstrate significant disparities in the shape, circularity, roundness, and aspect ratio of the particles comprising these injectables. This nuanced understanding may have critical implications for their mechanism of action and tissue interactions, thereby further elucidating their physiological effects and guiding clinical practice.

The primary morphological differences observed were in the shape of the particles. CaHA microspheres are smooth and homogeneous spherical particles with diameters ranging between 25 and 45 µm. In contrast, PLLA‐SCA microflakes displayed a mixture of irregularly shaped particles ranging from 2 to 150 µm in major axis length and support previous observations made on the irregularity of PLLA‐SCA particles.^17^, ^22^ Circularity and roundness measurements further highlighted differences between the two fillers. CaHA‐CMC microspheres demonstrated a significantly higher circularity and roundness compared to PLLA‐SCA particles. The tightly distributed circularity values of CaHA particles suggest a more uniform and circular shape, whereas PLLA‐SCA particles exhibited a broader distribution, indicating greater irregularity and heterogeneity. These findings were additionally supported by the AR results demonstrating CaHA‐CMC microspheres had an AR close to 1, consistent with a predominantly circular shape, while PLLA‐SCA particles had a significantly higher AR, indicating their oblong nature. This difference in AR further supports the notion that CaHA‐CMC microspheres possess a more uniform and predictable morphology compared to PLLA‐SCA particles.

**FIGURE 8:**
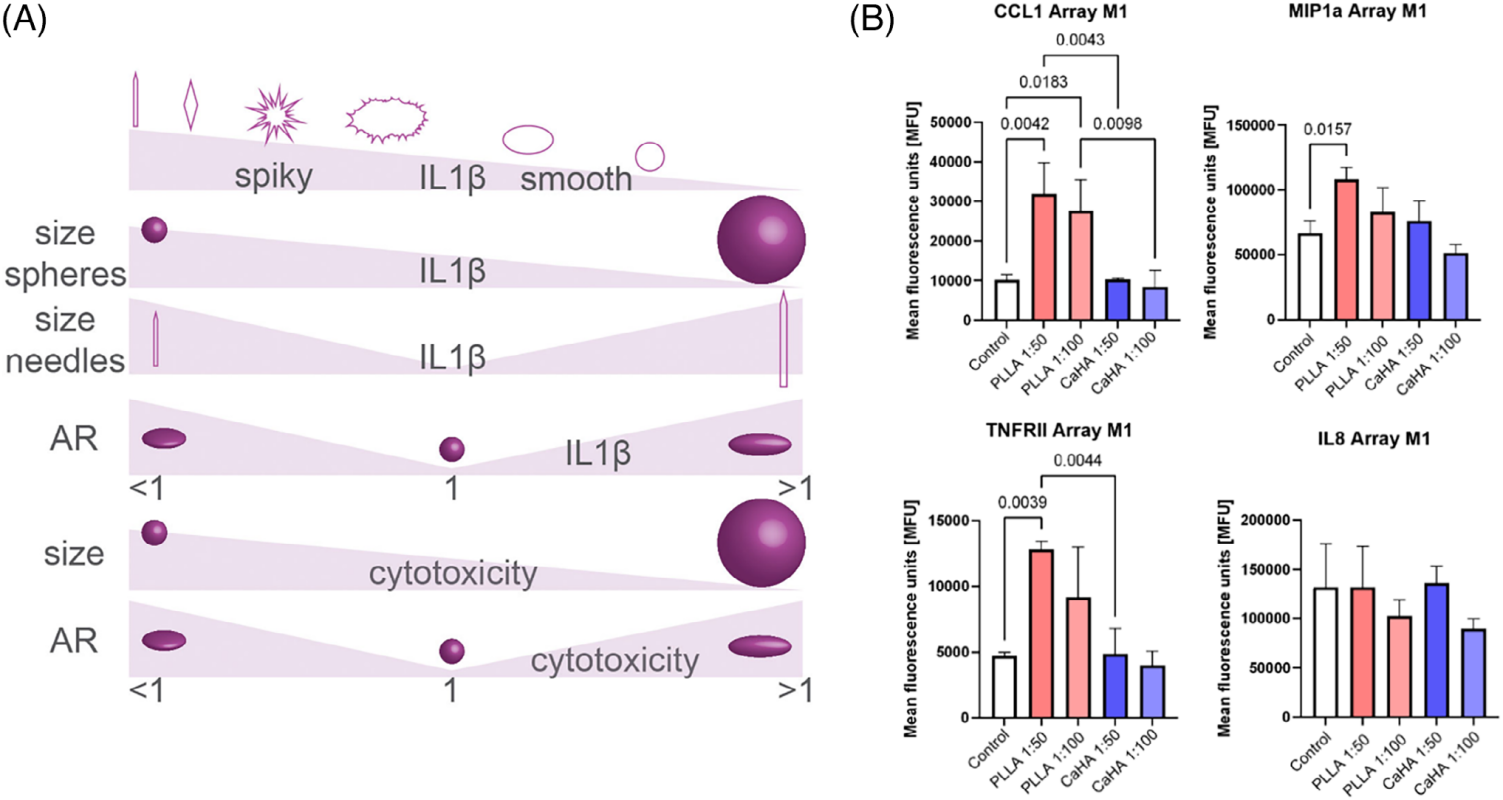
(A) Summary of particle geometry in inflammasome (IL‐1β production) and cytotoxicity. Reprinted from Baranov et al.^23^ (B) Cytokine levels in M1 macrophages after 24 h incubation with calcium hydroxylapatite (CaHA‐CMC) or PLLA identified with the human inflammation array. Adapted from Nowag et al.^18^

Particle morphology is arguably one of the most important determinants in material‐immune interactions. Descriptive physical features, such as circularity, roundness, and aspect ratio, are thought to significantly affect inflammasome activation. The body is known to recognize size and shape discrepancies in foreign objects, which subsequently modulate the immune response. Biomaterial properties and orientation have a significant effect on antigen presentation, with smooth, round, and spherical particles generally eliciting a less inflammatory response compared to spiky, pointy, rigid, or oblong counterparts. For instance, spikier particles are thought to disrupt phagosomal membranes leading to cathepsin leakage into the cytosol. That is, spikier particles are thought to disrupt phagosomal membranes leading to cathepsin leakage into the cytosol. Thus, spikier particles with low circularity and roundness, and particles with ARs deviating from 1 are thought to increase inflammasome activity (Figure 8A).^23^ For example, a study by Tabei et al., observed that phagocytosis of spiky calcium carbonate particles by macrophages resulted in increased IL‐8 and TNF‐α.^24^ This observation is supported by a recent study by Nowag et al., which co‐cultured M1 and M2 macrophages with diluted (1:50 and 1:100) particles of CaHA‐CMC and PLLA‐SCA, showing increases in pro‐inflammatory cytokines with PLLA‐SCA, including IL‐8 (Figure 8B). A plethora of similar studies, including one demonstrating microspherical hydroxyapatite particles are significantly less inflammatory than their spikey counterparts, exist showing similar findings.^25^, ^26^, ^27^

This principle of surface texture influencing the inflammatory response extends to the realm of breast implants. Textured implants, due to their rough and irregular surface, have been associated with a higher degree of inflammation and immune response compared to smooth implants. Britez et al., reported that textured breast implants induce a stronger local T‐cell immune response, potentially contributing to a higher incidence of capsular contracture and even the development of breast implant‐associated anaplastic large cell lymphoma.^28^

Aside from particle shape, their size is also of interest. If particles are too small (<20 µm), they are prone to easy phagocytosis, which may not only limit their duration of effect but also exacerbate inflammation. Conversely, overly large particles suffer from accumulation and lack of lateral tissue spread, potentially promoting nodule formation. Additionally, a combination of particles that vary significantly in size may result in sustained inflammation, increased degradation rates, and undesirable aesthetic outcomes. Factors such as particle size homogeneity, distribution, surface smoothness, roundness, and defects all play crucial roles in modulating the immune response and subsequent tissue interactions. CaHA‐CMC and PLLA‐SCA particles are generally intended to be between 25–45 µm and 10–200 µm, respectively, in major axis length, however, their fragments may be much smaller. The presence of fragments likely results from manufacturing and injection. Notably, CaHA is an extremely strong bioceramic that undergoes minimal deformation during injection.^3^ Comparatively, polymers such as PLLA are significantly more ductile and less tough than ceramics, meaning they may deform under lower shearing force, but likely do not break.^29^ Therefore, it is likely fragmentation is a result of manufacturing rather than injection. Cellularly, it is thought that smaller particle fragments (1–20 µm) are easily phagocytosed by macrophages.^21^, ^23^ These cellular findings have observed clinical impacts in aesthetic medicine, as macrophages undergoing phagocytosis of such foreign bodies release pro‐inflammatory cytokines that can, in turn, modulate macrophage subtype polarization and ECM remodeling.^30^ Lemperle noted that irregularly shaped particles have larger surface areas and less uniform surfaces and stimulate the release of cytokines from macrophages to a greater extent than spherical particles.^31^ In an observational human study, Lemperle also noted that 3 months after injection, PLLA was surrounded by macrophages and lymphocytes and by 6 months, was surrounded by macrophages and giant cells. In the same study subject, it was noted that CaHA‐CMC stimulated almost no foreign body reaction.^32^ Finally, Lemperle et al., posited that microparticles in aesthetic injectables must be free of phagocytosable particles under 20 µm.^21^ It was hypothesized that smaller fragments of particles may contribute to granuloma formation or delayed onset nodule formation by macrophage attack, citing studies showing reduced granuloma rates with polymethylmethacrylate fillers following the introduction of sieving and washing of microspheres. In this study, the percentage of phagocytosable particles (<20 µm), was significantly higher in PLLA‐SCA compared to CaHA.

Thus, it is conceivable that macrophage activation is greater with PLLA‐SCA than CaHA‐CMC and may explain differences in mechanism of action, with PLLA‐SCA having an immune‐mediated regenerative effect driven primarily by M2 macrophage polarization and CaHA‐CMC having a mechanotransductive effect independent of robust inflammation.^4^, ^33^ This hypothesis is supported by a recent gene expression study that showed PLLA‐SCA's mechanism of action largely functioned through the upregulation of inflammasomes IL1‐α, IL1‐β, and CXCL6, which contributed to dermal thickening and neocollagenesis,^16^ a study showing macrophage‐dependent neocollagenesis with PLLA‐SCA^11^, and studies showing contact‐dependent neocollagenesis with CaHA‐CMC.^4^ The body's ability to recognize size discrepancies in foreign objects underscores the importance of understanding and optimizing particle morphology to minimize adverse immune reactions and enhance therapeutic efficacy.

## 5 CONCLUSION

In conclusion, this study offers a detailed morphological comparison between CaHA‐CMC and PLLA‐SCA. The results reveal significant differences in the shape, circularity, roundness, and aspect ratio of the particles comprising these injectables. CaHA‐CMC demonstrated homogenous, smooth, and spherical CaHA microspheres, while PLLA‐SCA exhibited irregularly shaped PLLA‐SCA microflakes. The observed morphological variations between the two fillers could have important implications for their physiological effect. CaHA‐CMC's more uniform and spherical particles may contribute to the lack of observed inflammatory cell recruitment observed in histological studies, indicating a mechanotransductive mechanism of action. In contrast, PLLA‐SCA's irregularly shaped particles and higher percentage of phagocytosable particles could potentially lead to a more pronounced inflammatory response, indicating a macrophage‐mediated biostimulatory pathway. Further in vitro and in vivo studies analyzing the impact of CaHA‐CMC and PLLA‐SCA particles on inflammation and regeneration are warranted based on these results and the existing body of literature.

## CONFLICT OF INTEREST STATEMENT

McCarthy and Hartmann are employed by Merz Aesthetics. Shahriar and Xie have no conflicts to disclose. Durkin is a paid consultant, speaker, and trainer for Merz Aesthetics, Suneva, Apyx Medical,Babor, GloPharma, Alastin, Allergan, and the MusculoSkeletan Transplant Foundation. Khalifian is a paid consultant, speaker, trainer, and researcher for Allergan Aesthetics, Benev, Sciton, and Merz Aesthetics.

## Data Availability

Not available.
